# Supplementary material for: The phased pan-genome of tetraploid European potato
Source: Nature. 2025 Apr 16;642(8067):389–97. doi: 10.1038/s41586-025-08843-0 (PMC12158759; doi:10.1038/s41586-025-08843-0)
Supplement: Supplementary file 5 — Supplementary Figs. 3–14, 20, 21, 23–27 and 34–44. [file 41586_2025_8843_MOESM5_ESM.zip › suppl_figure_21/suppl_figure_21j_chr10.pdf]

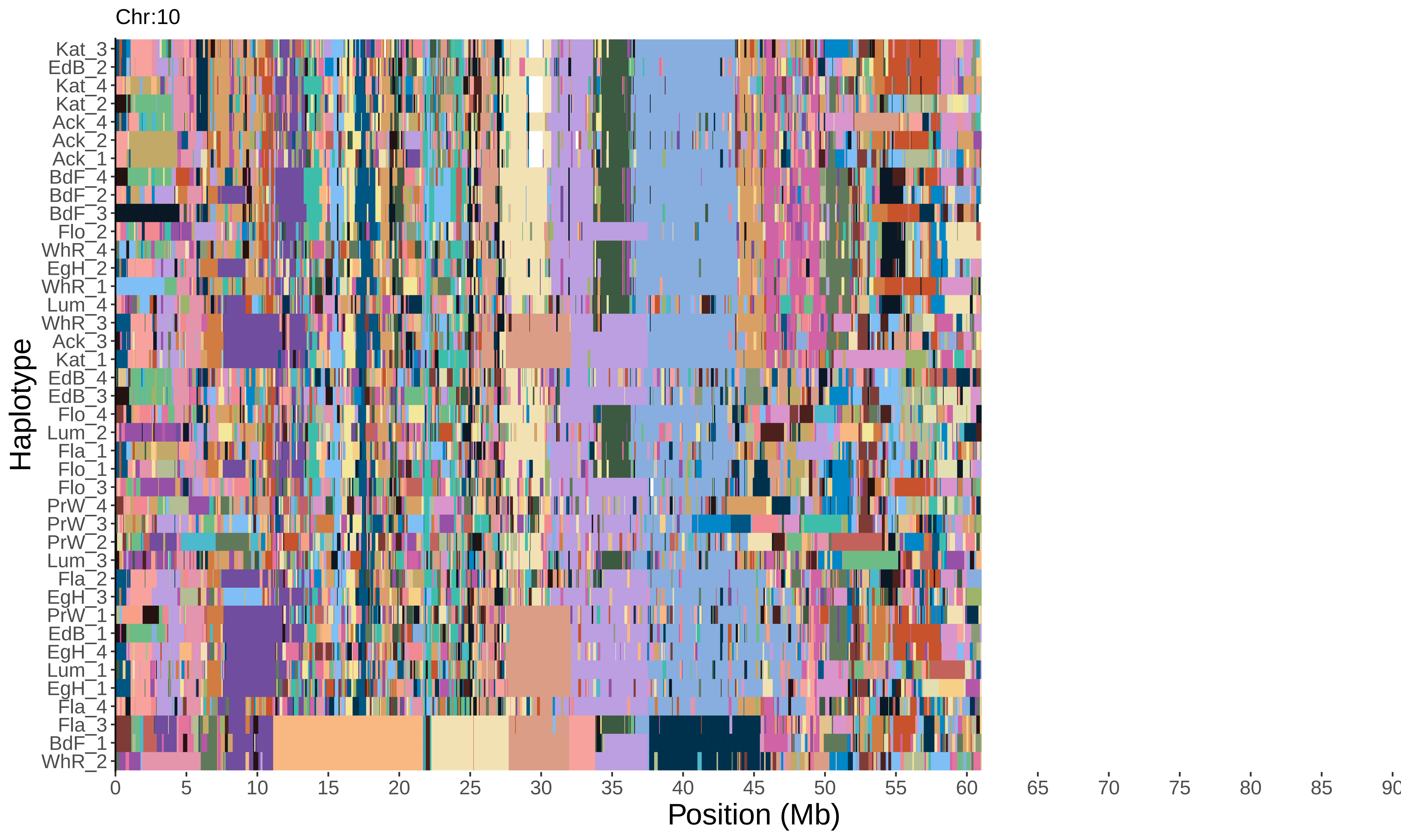
Chr:10
Kat_3
EdB_2
Kat_4
Kat_2
Ack_4
Ack_2
Ack_1
BdF_4
BdF_2
BdF_3
Flo_2
WhR_4
EgH_2
WhR_1
Lum_4
WhR_3
Ack_3
Kat_1
EdB_4
EdB_3
Flo_4
Lum_2
Fla_1
Flo_1
Flo_3
PrW_4
PrW_3
PrW_2
Lum_3
Fla_2
EgH_3
PrW_1
EdB_1
EgH_4
Lum_1
EgH_1
Fla_4
Fla_3
BdF_1
WhR_2
Haplotype
0 5 10 15 20 25 30 35 40 45 50 55 60 65 70 75 80 85 90
Position (Mb)
